# Complete Transverse Division of the Urethra by a Kick of a Horse on the Perineum, &c.

**Published:** 1867-05

**Authors:** 


*Complete Transverse Division of the Urethra by a Kick of a Horse on the Perineum; Perineal Section; Recovery.* Under the care of Mr. Birkett.

G. C——, aged fourteen, a fine healthy youth, was admitted at midday on June 28th, 1866. About twenty-four hours previously a horse kicked him in the perineum. He did not feel much pain at the time, and walked home, a distance of a quarter of a mile, immediately afterwards. He then went to bed. A medical man visited him in the evening, and endeavored to pass a catheter, but without success. Warm fomentations were then applied to the abdomen, and some blood flowed from the urethra. In the night he felt pain in the loins. Next morning, having passed no urine since the accident, he was brought to the hospital.

On admission, the perineum was slightly swollen and ecchymosed; the bladder, much distended, reached far above the pubes. Unsuccessful attempts were made by Mr. Birkett to pass a catheter into the bladder, the point of the instrument being arrested in its course just beneath the bubic arch. Considerable hæmorrhage from the urethra accompanying these gentle attempts to pass a catheter led Mr. Birkett to the conclusion that the canal was torn; more particularly, too, as the instrument seemed disposed to take an erratic course, and its point could be felt *too* distinctly in the perineum. He decided therefore to open the perineum. Chloroform was given; and after another unsuccessful attempt to introduce a flexible catheter, a grooved staff was passed down to the perineum, and an incision made along the raphé from the posterior edge of the scrotum to the anus for about an inch and a half. Some coagulated blood was turned from the depths of the wound, when the urethra and its surrounding textures were found completely divided transversely. By compressing the bladder over the pubes, urine was made to issue from the posterior division of the ruptured canal, which was hanging into the wound, in consequence of its being detached from the corpora cavernosa, for about a quarter of an inch. The line of rupture in the urethra was not perfectly transverse, but ran obliquely across from below anteriorly to above at the back part; and, in consequence of the contractility of the tissue, the two ends of the divided canal were fully half an inch apart. Mr. Birkett passed a flexible catheter along the anterior division of the urethra, out at the perineal wound, and thence through the posterior division into the bladder; he then brought together the two ends of the ruptured urethra upon the catheter, and tied them with one silk suture introduced in the middle line of the under surface. The urethra was not touched by the knife during the operation. The flexible catheter was fixed in the bladder.

June 30th (second day).—All the urine passed through the catheter; none by the perineal wound. The skin around the wound was healthy, and the sides of the wound were bathed with pus. He slept and ate well, and had no pain.

July 6th (eighth day).—Catheter removed from urethra; it was slightly coated at the end with phosphates.

7th (ninth day).—Since the removal of the instrument he had made water four times; it mostly passed in the proper direction, but a little escaped at the perineal wound (less latterly than at first). The urine first passed was turbid and contained albumen.

9th (eleventh day).—Urine escaped freely through perineum; its passage unaccompanied with smarting. The urine was not albuminous.

12th (fourteenth day).—Made water easily; very little passing through perineum.

26th (twenty-eighth day).—The quantity of urine coming by the perineum had gradually decreased until, during the three prceding days, there had not been any escape in that direction. The wound was healed. He had never had a bad symptom; and he left the hospital to-day, having been under treatment twenty-eight days. The suture was never seen, although it was probably cast off in the discharge.

November 8th.—He called on Mr. Birkett to-day. He passed a full stream of urine without the sligtest difficulty; and there seemed to be no symptom of contraction of the urethra, although three months had elapsed since the perineal wound was healed.—*Ib.*

